# Relationship with Grandparents and Young Adults’ Attitudes About Aging and Future-Oriented Tendencies

**DOI:** 10.1093/geroni/igaa057.3260

**Published:** 2020-12-16

**Authors:** Tianyuan Li, Pok Man Siu

**Affiliations:** Education University of Hong Kong, Hong Kong, China

## Abstract

It is important but always challenging to restrain from immediate temptations and behave conscientiously for long-term goals. Constructive interactions with older adults may promote young adults’ positive attitudes about aging. With a brighter later adulthood in mind, young adults may then demonstrate a higher level of future-oriented tendency in their behaviors. The current study recruited 371 college students (Mage = 22.43, SDage = 2.88; 203 females) from Hong Kong. They completed an online survey about their interaction with the closest grandparent, attitudes about aging, and measures related to future-oriented tendencies (i.e., consideration of future consequences, healthy lifestyle, and impulsiveness). Parental intimacy and demographic information were assessed as potential covariates. Supporting the hypotheses, more interaction with grandparents was related to more positive attitudes about aging, β = .40, SE = .11, p < .001. More positive attitudes about aging was then related to more consideration of future consequences, β = .14, SE = .07, p = .03, healthier lifestyle, β = .16, SE = .06, p = .005, and less impulsivity, β = -.10, SE = .03, p < .001. Bootstrapping tests for the indirect effects from interaction with grandparents to the future-oriented outcomes through positive attitudes about aging were all significant as well. Although the current cross-sectional data could not confirm the causal links among the variables, the results provide some initial insight on how older adults can foster a long-term orientation in younger generations and contribute to the sustainable development of our societies through constructive intergenerational interactions.

